# A Single Dose of Anti-HBsAg Antibody-Encoding mRNA-LNPs Suppressed HBsAg Expression: a Potential Cure of Chronic Hepatitis B Virus Infection

**DOI:** 10.1128/mbio.01612-22

**Published:** 2022-07-07

**Authors:** Binfan Chen, Yuchen Chen, Jian Li, Chunyu Wang, Wenping Song, Yumei Wen, Jinzhong Lin, Yanling Wu, Tianlei Ying

**Affiliations:** a MOE/NHC/CAMS Key Laboratory of Medical Molecular Virology, Shanghai Institute of Infectious Disease and Biosecurity, Shanghai Engineering Research Center for Synthetic Immunology, School of Basic Medical Sciences, Fudan University, Shanghai, China; b State Key Laboratory of Genetic Engineering, School of Life Sciences, Zhongshan Hospital, Fudan University, Shanghai, China; c Clinical Laboratory Center, Children's Hospital of Fudan University, National Children's Medical Center, Shanghai, China; University of Calgary

**Keywords:** HBV infection, HBsAg, functional cure, immune response, antibody, mRNA, CHB

## Abstract

Hepatitis B virus (HBV) infection is a serious global health issue with more than 250 million chronic carriers. It causes liver diseases such as chronic hepatitis, liver cirrhosis, and hepatocellular carcinoma (HCC). Persistent suppression of the HBV surface antigen (HBsAg) is necessary for a functional cure of chronic hepatitis B (CHB) virus infection. However, this can hardly be achieved with currently approved drugs. Antibody treatment against HBsAg has shown promise in restoring HBV-specific immune responses and promoting HBV cure. To achieve long-lasting HBsAg suppression, we used an advanced mRNA drug to encode the genes of three anti-HBsAg antibodies, G12-scFv, G12-scFv-Fc, and G12-IgG. Antibody-encoding mRNA-lipid nanoparticles (LNPs), mL (G12-scFv-Fc) and mL (G12-IgG), substantially reduced serum HBsAg levels in treated mice within 30 days after a single dose. In contrast, exogenous antibodies lost effect on reducing HBsAg or HBV DNA levels 9 days postadministration. The high affinity of anti-HBsAg antibodies and the adjuvant activity of mRNA-LNPs resulted in long-term HBsAg seroclearance, which could contribute to the reestablishment of the immune system in HBV carriers. These findings highlight the great potential of antibody-encoding mRNA molecules against CHB infection.

## INTRODUCTION

Therapeutic antibodies have become the predominant treatments for various pathological states such as cancers and autoimmune and metabolic diseases (1). Compared to vaccines, antibodies show pivotal potential modalities for diagnosis, short-term prevention, and treatment of infectious diseases (2). Antibodies provide very rapid protection from infectious diseases, while vaccines usually need a longer time and several doses to induce an effective immune response. Furthermore, passive immunization could prevent or protect against infections in healthy individuals and immune-deficient patients (3). However, the process of antibody manufacturing is complex, expensive and time-consuming, limiting the application of antibodies in infections.

By intramuscular injection of mRNA into mice, Wolff et al. (4) found, for the first time, in 1990 that encoded antigen expression and immune response against the antigen could be detected. *In vitro*-transcribed mRNA (IVT-mRNA) synthesis (5–7), chemical modification (8, 9), and delivery technology (10, 11) have been developed for decades, which made it possible to apply mRNA technology to vaccine research. By 2020, effective vaccines were urgently needed to control the global COVID-19 pandemic. As a genetic format, mRNA vaccines may address the shortcomings of current vaccine technologies. Without bacterial or cell culture requirements, mRNA-based vaccines offer a fast, cost-effective, and standardizable approach to SARS-CoV-2 vaccine development.

Moreover, mRNA vaccines could induce humoral and cellular immune responses and have adjuvant roles in activating immune responses. Under this circumstance, the Pfizer/BioNTech and Moderna mRNA vaccines were approved for widespread use. Also, they have been proven significantly more effective than an inactivated vaccine against COVID-19 (12). mRNA vaccines are also effective against many other acute infections such as Zika (13) and influenza (14) and chronic infections such as HIV (15). However, only a few preclinical studies of passive immunization by mRNA-encoded antibodies have been reported (16–18). One study of Chikungunya protection has been moved into a phase I clinical trial (ClinicalTrials registration no. NCT03829384). Therefore, passive immunization with antibody-encoding mRNA is a novel protective strategy for virus infections that deserves further investigation.

More than 257 million patients worldwide suffer from chronic hepatitis B (CHB) virus infection (19). Hepatitis B virus (HBV) dramatically changes antibody repertoires of CHB-infected individuals (20), induces defective T-cell and B-cell function, and causes persistent HBV infection (21), which could progress to cirrhosis and, finally, lead to liver failure and hepatocellular carcinoma (HCC) (22). The impaired host immune system and the permanent existence of covalently closed circular DNA (cccDNA) are the main obstacles to HBV clearance, making the complete cure inaccessible. A functional cure of CHB, defined by the loss of the HBV surface antigen (HBsAg), is attainable and the primary goal of current treatment for CHB. Currently, the valid CHB therapies are nucleos(t)ide analogs, interferon, and PEGylated interferon, which only result in less than 10% functional cure from HBV infections due to the limited therapeutic outcomes (22, 23). Various therapies targeting innate immunity and HBV-specific immune responses are under clinical investigation (24). Among these medications, anti-HBsAg antibodies are potent candidates (25–27). Antibody therapies against HBsAg remarkably reduced circulating HBsAg and HBV DNA levels in several mouse models and restored adaptive immunity functionality that was defective due to CHB. However, anti-HBsAg antibodies only suppressed HBsAg within a finite duration (26).

To deal with such a problem, we used anti-HBsAg antibody-encoding mRNA-lipid nanoparticles (LNPs) in our study. Three anti-HBsAg antibodies (G12-scFv, G12-scFv-Fc, and G12-IgG) were encoded by mRNA and encapsulated in LNP. The mRNA-LNP of G12 provided persistent passive immunization for at least 30 days. Moreover, mRNA was mainly delivered to the hepatocytes by LNP (28). These characteristics made anti-HBsAg antibody-encoding mRNA-LNPs promising for the functional cure of HBV.

## RESULTS

### Design of G12 mRNA.

**FIG 1:**
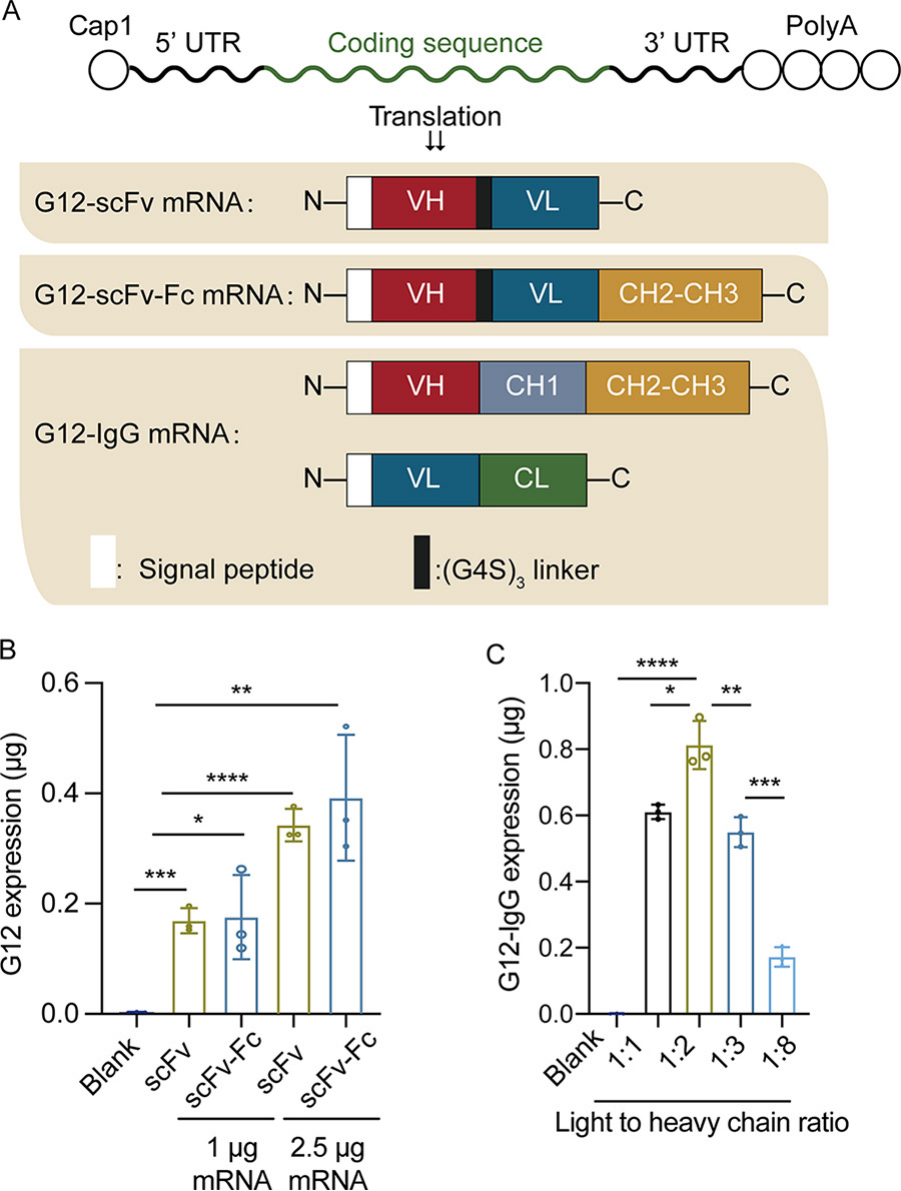
Preparation of G12 IVT mRNAs. (A) Design of four mRNAs for the expression of G12-scFv, G12-scFv-Fc, and G12-IgG. (B) Expression levels of G12-scFv and G12-scFv-Fc IVT-mRNA in 293T cells. (C) G12-IgG levels in 293T cells medium 24 h after IVT-mRNA transfection with LC mRNA to HC mRNA at 1:1, 1:2, 1:3, and 1:8 ratios.

Four mRNAs, scFv mRNA, G12-scFv-Fc mRNA, G12 heavy-chain (HC) mRNA, and G12 light-chain (LC) mRNA, were synthesized. Each mRNA comprised a 5′ cap1 structure, 5′ untranslated region (UTR), coding sequence, 3′ UTR, and a poly(A) tail (Fig. 1A). G12-scFv mRNA was used to express G12-scFv, and G12-scFv-Fc mRNA was for G12-scFv-Fc production. G12 HC mRNA and G12 LC mRNA were used together to obtain G12-IgG. For G12-scFv and G12-scFv-Fc expression, the level of antibodies were elevated about twice when mRNA dosages increased from 1 μg to 2.5 μg per 2 × 10^5^ 293T cells (Fig. 1B). To optimize the expression of G12-IgG, LC mRNA and HC mRNA were cotransfected. We screened four mass ratios of LC mRNA to HC mRNA to optimize the yield of G12-IgG. The highest expression of G12-IgG was obtained when the mass ratio of LC mRNA to HC mRNA was 1:2 (Fig. 1C). This result was consistent with the previous research on trastuzumab production (29). Thus, the three G12 antibodies could be successfully produced *in vitro* using mRNAs. In our study, the mass ratio of 1:2 (LC mRNA to HC mRNA) was applied for G12-IgG production.

### *In vivo* confirmation of G12 antibody expressions.

**FIG 2:**
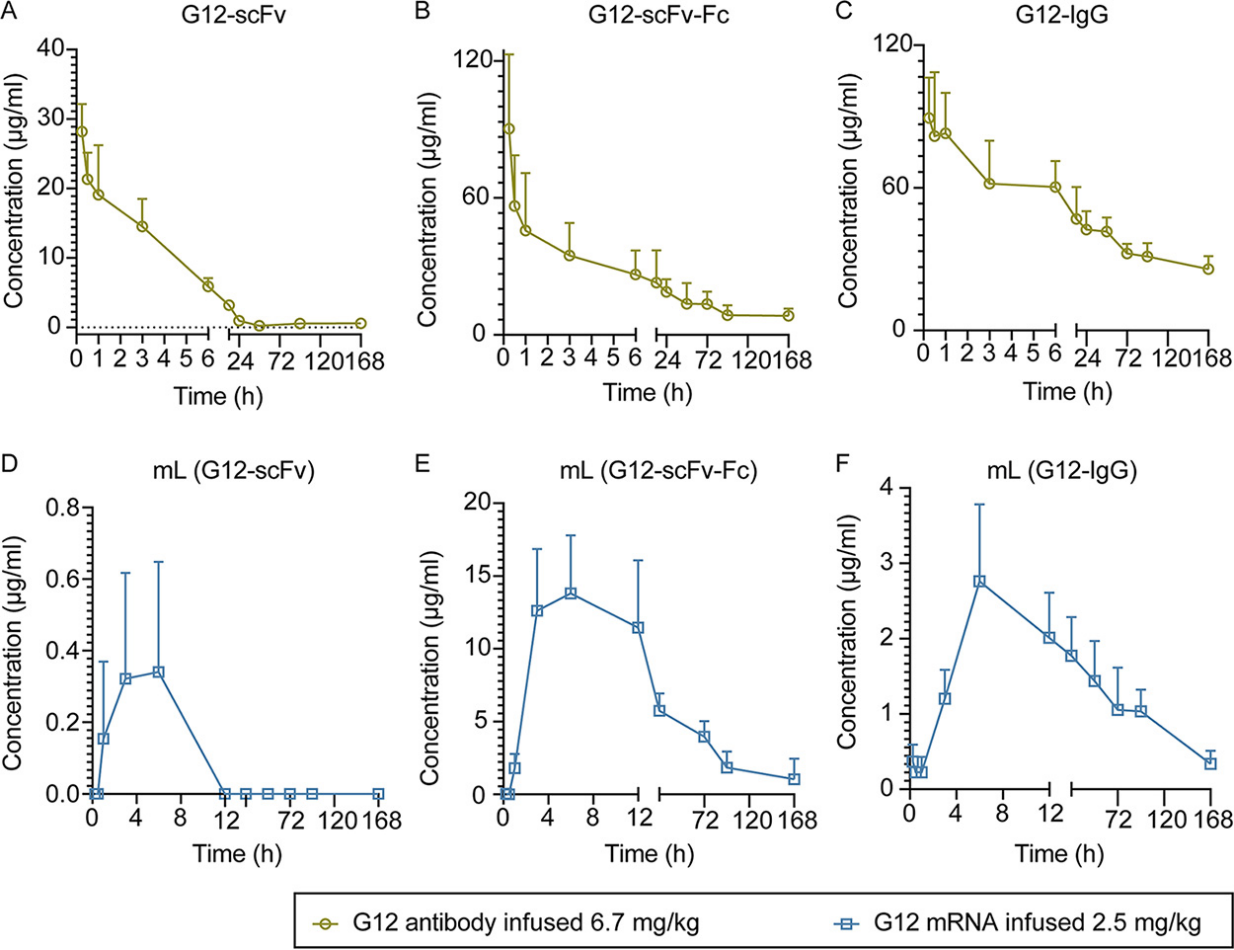
Pharmacokinetic characterization of G12 antibody *in vivo* after i.v. administration of antibodies or mRNA-LNPs. The remaining G12 antibodies in the serum were monitored for 7 days. (A) G12-scFv; (B) G12-scFv-Fc; (C) G12-IgG; (D) mL (G12-scFv); (E) mL (G12-scFv-Fc); (F) mL (G12-IgG).

To investigate the production of G12 antibody *in vivo*, we prepared three mRNA-LNPs, mL (G12-scFv), mL (G12-scFv-Fc), and mL (G12-IgG), with particle sizes of 149.6 nm, 154.0 nm, and 186.1 nm and zeta potentials of +13.0 mV, +13.8, and +14.3 mV, respectively (see Fig. S1 in the supplemental material). The encapsulation efficiencies were higher than 85%. LNPs were intravenously administered to C57 mice at a dose of 2.5 mg/kg body weight. The antibodies of G12-scFv, G12-scFv-Fc, and G12-IgG were intravenously administered to C57 mice at 6.7 mg/kg. Mice injected with antibodies attained maximum concentrations (peak serum concentration [*C*_max_]) 15 min after administration. We detected 28.9 ± 2.6 μg/mL G12-scFv, 90.3 ± 32.6 μg/mL G12-scFv-Fc, and 95.3 ± 20.5 μg/mL G12-IgG in the serum, respectively (Fig. 2A to C). The concentrations dramatically dropped in subsequent observations. On the other hand, the three G12 antibodies encoded by mRNAs reached peak concentrations (0.6 ± 0.2 μg/mL G12-scFv, 14.2 ± 3.8 μg/mL G12-scFv-Fc, and 2.4 ± 1.24 μg/mL G12-IgG) in the mouse serum at different times postadministration (Fig. 2D to F). The level of G12-scFv in serum was lower than G12-scFv-Fc and G12-IgG, consistent with mice administered by antibody directly and by mRNA-LNP, indicating the fast clearance of G12-scFv *in vivo*. IgG antibodies showed the highest antibody concentration when the mice were injected with antibodies. However, mL (G12-scFv-Fc) provided a higher antibody expression level than other LNPs. As shown in Fig. 2E, a high serum level of G12-scFv-Fc lasted for about 8 h.

10.1128/mbio.01612-22.1 FIG S1 Characteristics of G12 IVT-mRNA-LNP. Particle size distribution of mL (G12-scFv) (A), mL (G12-scFv-Fc) (C), and mL (G12-IgG) (E). Zeta potential distribution of mL (G12-scFv) (B), mL (G12-scFv-Fc) (D), and mL (G12-IgG) (F). Download FIG S1, TIF file, 0.2 MB. Copyright © 2022 Chen et al. 2022 Chen et al. https://creativecommons.org/licenses/by/4.0/ This content is distributed under the terms of the Creative Commons Attribution 4.0 International license.

The serum drug level dropped sharply soon after the administration of the antibody. In contrast, injection of mRNA-LNP gave a persistent antibody expression *in vivo*. Antibodies were consecutively synthesized unless the mRNA was degraded. The half-time of endogenous antibodies was positively correlated with that of mRNA (Table 1). As shown in Fig. 2D through F, endogenous antibodies accumulated during the first 4 h after administration, and serum antibody was maintained at a high level for more than 8 h. These results indicated that mRNA-LNP was a promising platform for *in vivo* production of G12 antibodies.

**TABLE 1:** Analysis of G12 pharmacokinetics in mouse serum after a single i.v. dose of G12 antibodies or antibody-encoding mRNA-LNPs^*a*^

Treatment	*t*_1/2_ (h)	AUC (μg/mL·h)
G12-scFv	8.7 ± 3.9	156.4 ± 17.3
G12-scFv-Fc	88.8 ± 37.9	2,326.9 ± 942.3
G12-IgG	200.3 ± 186	5,948.9 ± 745.1
mL (G12-scFv)		
mL (G12-scFv-Fc)	48.3 ± 11.4	661.1798 ± 196.3
mL (G12-IgG)	58.3 ± 15.2	160.7 ± 79.1

a Calculations were performed using Phoenix WinNonLin. *t*_1/2_, half-life; AUC_0–_*_t_*, area under the concentration-time curve.

### Evaluation of *in vivo* efficacy of mL (G12-scFv) in the AAV/HBV mouse model.

**FIG 3:**
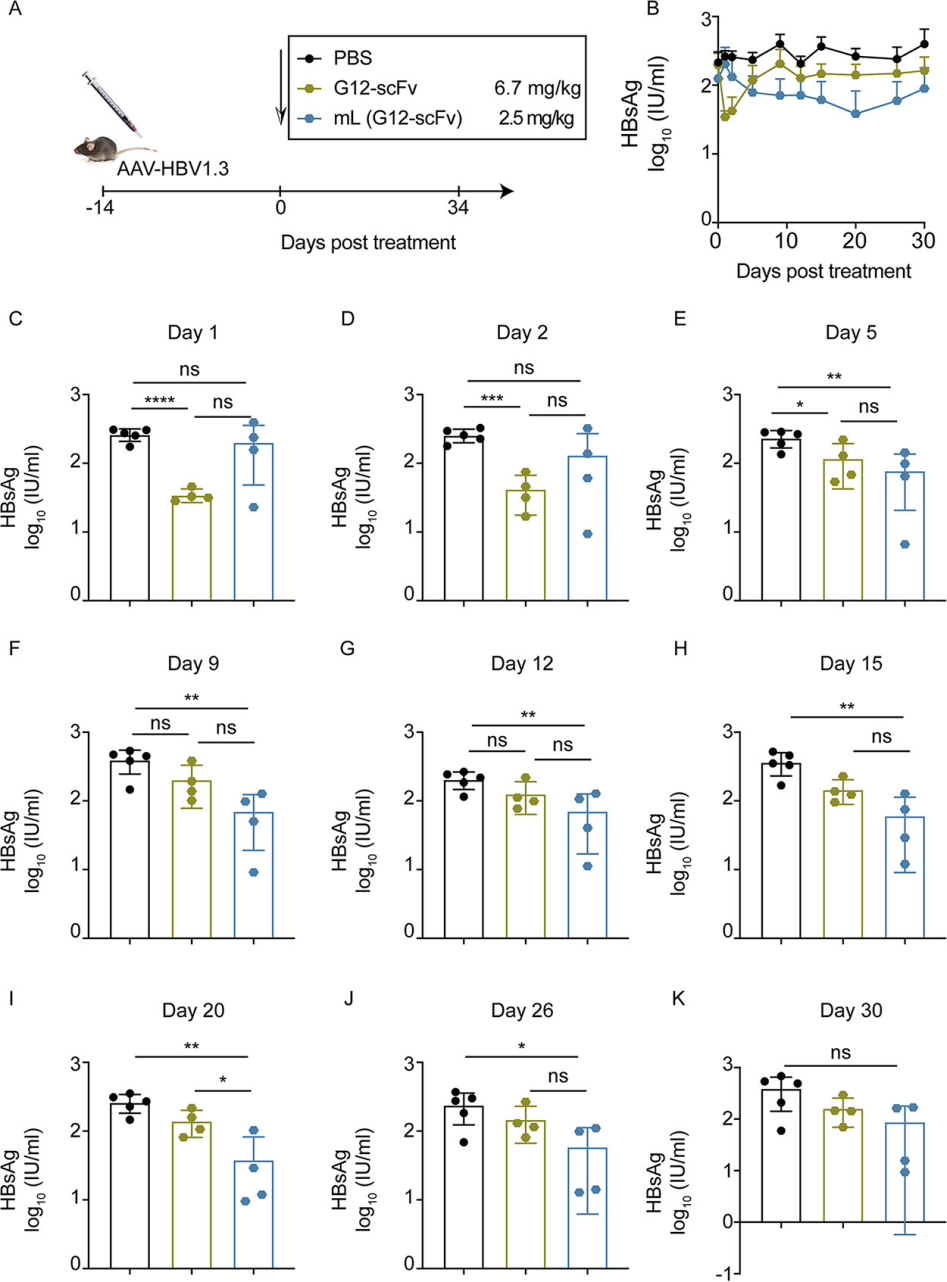
mL (G12-scFv) decreased HBsAg in AAV/HBV mouse model. (A) AAV/HBV mice were injected with 2.5 mg/kg mL (G12-scFv) or 6.7 mg/kg G12-scFv. (B) Kinetics of HBsAg levels in 30 days posttreatment. HBsAg levels were quantified on day 1 (C), day 2 (D), day 5 (E), day 9 (F), day 12 (G), day 15 (H), day 20 (I), day 26 (J), and day 30 (K) posttreatment.

To evaluate the efficacy of mRNA (G12)-LNP, the adeno-associated virus (AAV)/HBV mouse model with persistent HBsAg expression was established. Mice were intravenously (i.v.) injected with single dose of either 2.5 mg/kg mL G12-scFv or 6.7 mg/kg G12-scFv (Fig. 3A). Although the serum HBsAg was slightly downregulated, their changes were relatively mild following administration of mL (G12-scFv) or G12-scFv (Fig. 3B). G12-scFv reduced HBsAg levels by approximately 0.5 log_10_ in mouse serum during the first 2 days (Fig. 3C and D). HBsAg restoration emerged on day 5 (Fig. 3E), and after that, G12-scFv no longer contributed to HBsAg seroclearance (Fig. 3E through K). On the contrary, mL (G12-scFv) barely altered serum HBsAg levels in the first 2 days (Fig. 3C and D), and from day 5 onwards, it persistently reduced HBsAg levels by over 0.5 log_10_ for more than 15 days (Fig. 3E through K).

As expected, G12-scFv significantly reduced serum HBV DNA levels only in the first 2 days (*P < *0.05) (Fig. S2A through D). In contrast, serum HBV DNA levels in LNP-treated mice reduced after day 2 (*P < *0.05) (Fig. S2C through D). On day 5, the lowest level of HBV DNA (2.7 log_10_) in serum was observed (*P < *0.01) (Fig. S2E through K). Compared to G12-scFv, mL (G12-scFv) provided a more sustained effect.

10.1128/mbio.01612-22.2 FIG S2 mL (G12-scFv) decreased HBV DNA in an AAV/HBV mouse model. (A) AAV/HBV mice were injected with 2.5 mg/kg mL (G12-scFv) or 6.7 mg/kg G12-scFv. (B) Kinetics of HBV DNA levels in 30 days posttreatment. HBV DNA levels were quantified on day 1 (C), day 2 (D), day 5 (E), day 9 (F), day 12 (G), day 15 (H), day 20 (I), day 26 (J), and day 30 (K) posttreatment. Download FIG S2, TIF file, 0.5 MB. Copyright © 2022 Chen et al. 2022 Chen et al. https://creativecommons.org/licenses/by/4.0/ This content is distributed under the terms of the Creative Commons Attribution 4.0 International license.

Neither mL (G12-scFv) nor G12-scFv had a significant effect on HBsAg clearance, despite the statistical difference from the untreated group. This result was due to the low expression of G12-scFv after administration of mL (G12-scFv) and the rapid clearance and lower 50% effective concentration (EC_50_) of G12-scFv than that of G12-scFv-Fc and G12-IgG (Fig. S5).

10.1128/mbio.01612-22.5 FIG S5 Affinity of G12 antibodies to HBsAg. EC_50_ determinations of G12-scFv (A), G12-scFv-Fc (B), and G12-IgG (C). Download FIG S5, TIF file, 0.1 MB. Copyright © 2022 Chen et al. 2022 Chen et al. https://creativecommons.org/licenses/by/4.0/ This content is distributed under the terms of the Creative Commons Attribution 4.0 International license.

### Evaluation of *in vivo* efficacy of mL (G12-scFv-Fc) in the AAV/HBV mouse model.

**FIG 4:**
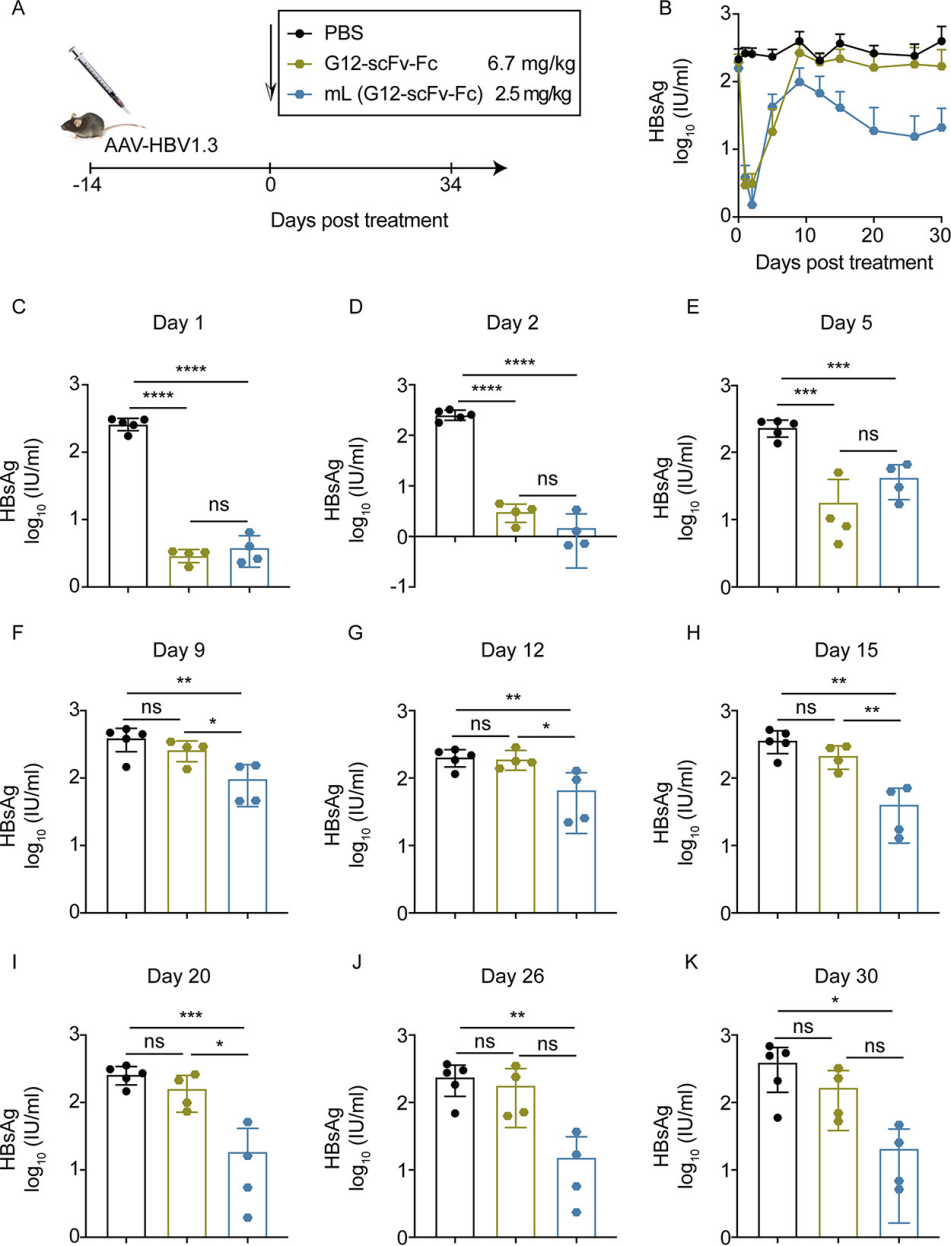
mL (G12-scFv-Fc) decreased HBsAg in AAV/HBV mouse model. (A) AAV/HBV mice were injected with 2.5 mg/kg mL (G12-scFv-Fc) or 6.7 mg/kg G12-scFv-Fc. (B) Kinetics of HBsAg levels at 30 days posttreatment. HBsAg levels were quantified on day 1 (C), day 2 (D), day 5 (E), day 9 (F), day 12 (G), day 15 (H), day 20 (I), day 26 (J), and day 30 (K) posttreatment.

Similarly, 2.5 mg/kg mL (G12-scFv-Fc) or 6.7 mg/kg G12-scFv-Fc was administered (Fig. 4A). During 30 days of monitoring, G12-scFv-Fc was only effective on HBsAg seroclearance within 5 days after administration, while mL (G12-scFv-Fc) provided a constant reduction in serum HBsAg levels for 30 days (Fig. 4B). In detail, G12-scFv-Fc and mL (G12-scFv-Fc) both resulted in extremely low serum HBsAg levels for 2 days (*P < *0.0001) (Fig. 4C and D), followed by a rebound on day 5 (*P < *0.001) (Fig. 4E). From day 9, G12-scFv-Fc did not affect HBsAg elimination (Fig. 4F through K). In contrast, mL (G12-scFv-Fc) decreased serum HBsAg levels and reached the plateau phase on day 20 (Fig. 4G through K). G12-scFv-Fc dramatically reduced HBV DNA levels within the first 5 days (*P < *0.05) (Fig. S3A through K). mL (G12-scFv-Fc) significantly reduced the HBV DNA level on day 2 (*P < *0.05) and day 5 (*P* < 0.01) (Fig. S3D and E). Also, it is worth noticing that mL (G12-scFv-Fc) restored the effect of serum HBV DNA clearance on days 20 and 26 (*P < *0.01) (Fig. S3I through J).

10.1128/mbio.01612-22.3 FIG S3 mL (G12-scFv-Fc) decreased HBV DNA in AAV/HBV mouse model. (A) AAV/HBV mice were injected with 2.5 mg/kg mL (G12-scFv-Fc) or 6.7 mg/kg G12-scFv-Fc. (B) Kinetics of HBV DNA levels in 30 days posttreatment. HBV DNA levels were quantified on day 1 (C), day 2 (D), day 5 (E), day 9 (F), day 12 (G), day 15 (H), day 20 (I), day 26 (J), and day 30 (K) posttreatment. Download FIG S3, TIF file, 0.5 MB. Copyright © 2022 Chen et al. 2022 Chen et al. https://creativecommons.org/licenses/by/4.0/ This content is distributed under the terms of the Creative Commons Attribution 4.0 International license.

Unlike the short effect caused by G12-scFv-Fc, mL (G12-scFv-Fc) induced a persistent suppression of serum HBsAg and HBV DNA levels. Although the serum concentrations of G12-scFv-Fc in the G12-scFv-Fc group are higher than that in the mL (G12-scFv-Fc) group (Fig. 2B and E), mL (G12-scFv-Fc), rather than G12-scFv-Fc, reported considerable strong serum HBsAg reduction. These contradictory results still need more investigation.

### *In vivo* efficacy of mL (G12-IgG) in the AAV/HBV mouse model.

**FIG 5:**
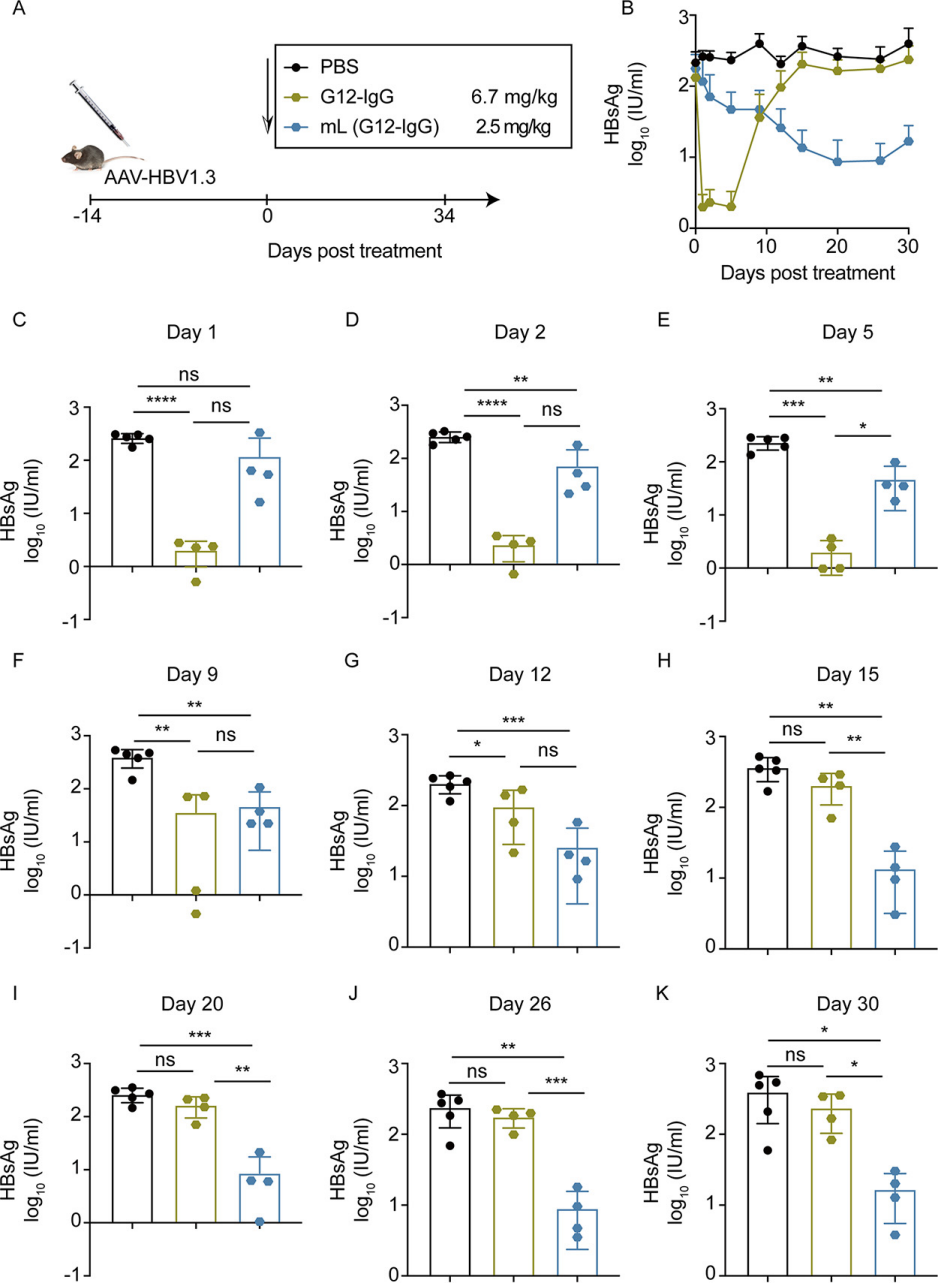
mL (G12-IgG) decreased HBsAg in an AAV/HBV mouse model. (A) AAV/HBV mice were injected with 2.5 mg/kg mL (G12-IgG) or 6.7 mg/kg G12-IgG. (B) Kinetics of HBsAg levels in 30 days posttreatment. HBsAg levels were quantified on day 1 (C), day 2 (D), day 5 (E), day 9 (F), day 12 (G), day 15 (H), day 20 (I), day 26 (J), and day 30 (K) posttreatment.

mL (G12-IgG) or G12-IgG was given to C57 mice at 2.5 mg/kg and 6.7 mg/kg, respectively (Fig. 5A). During the 30-day monitoring period, G12-IgG induced a drastic decrease and rapid rebound of serum HBsAg, while mL (G12-IgG) caused continuous enhancement in HBsAg seroclearance (Fig. 5B). As the results showed, the effect on HBsAg elimination of the G12 IgG was the highest on the first day and steadily went down over time as follows: day 2 (*P < *0.0001), day 5 (*P < *0.001), day 9 (*P < *0.01), day 12 (*P < *0.05), and day 15 (*P = *not significant) (Fig. 5C through H). With progressively increasing impacts on HBsAg reduction, mL (G12-IgG) lowered the serum HBsAg level by less than 1 log_10_ within 12 days (day 2 to day 9, *P < *0.01; day 12, *P < *0.001) (Fig. 5D through G) and by over 1 log_10_ on day 15 (Fig. 5H). On day 20, mL (G12-IgG) reached its bottom line of serum HBsAg levels (Fig. 5I). Noticeably, mL (G12-IgG) consecutively decreased the serum HBsAg levels from day 2 to day 30 (Fig. 5D through K).

G12-IgG reduced HBV DNA levels immediately after treatment (Fig. S4A through D), and the effect peaked on day 5 (*P < *0.01) (Fig. S4E), followed by a rebound (Fig. S4F through K). mL (G12-IgG) made serum HBV DNA levels plunge on day 2 (*P < *0.05) and day 5 (*P < *0.01) (Fig. S4D through E). It is noteworthy that mL (G12-IgG) provided a more significant and persistent suppression of serum HBV DNA levels on days 20 and 26 (*P < *0.05) (Fig. S4I through J). Furthermore, the HBV DNA-lowering effect achieved by mL (G12-IgG) was significantly greater than that by G12-IgG, regardless of whether the difference was significant.

10.1128/mbio.01612-22.4 FIG S4 mL (G12-IgG) decreased HBV DNA in AAV/HBV mouse model. (A) AAV/HBV mice were injected with 2.5 mg/kg mL (G12-IgG) or 6.7 mg/kg G12-IgG. (B) Kinetics of HBV DNA levels in 30 days posttreatment. HBV DNA levels were quantified on day 1 (C), day 2 (D), day 5 (E), day 9 (F), day 12 (G), day 15 (H), day 20 (I), day 26 (J), and day 30 (K) posttreatment. Download FIG S4, TIF file, 0.4 MB. Copyright © 2022 Chen et al. 2022 Chen et al. https://creativecommons.org/licenses/by/4.0/ This content is distributed under the terms of the Creative Commons Attribution 4.0 International license.

Although mL (G12-IgG) took a while to block HBsAg, it was impressive that it could exert an increasingly consistent effect on reducing the HBsAg level. mL (G12-IgG) exhibited a more persistent effect on HBV seromarkers despite the higher serum concentration provided by G12-IgG.

## DISCUSSION

HBsAg clearance has been recognized as a long-term goal of anti-HBV therapy. Due to the low efficiency of HBsAg downregulation and the high frequency of viral rebound (30), currently approved therapies for CHB, such as pegylated interferon and nucleoside analogs (NAs), fail to cure HBV completely (29). Anti-HBsAg antibodies are valuable serological indicators. They provide direct HBsAg suppression in clinical trials and have the potential for a functional cure of CHB (31–33). However, the widespread application of antibody therapy is hindered by the requirement for high dosages and repeat administration.

Our study used lipid nanoparticles (LNPs) to deliver mRNA encoding G12 antibodies *in vivo*. The mRNA-LNPs and antibodies for G12-scFv, G12-scFv-Fc, and G12-IgG were prepared *in vitro*. In terms of HBsAg serum clearance, G12-IgG had the best efficacy, followed by G12-scFv-Fc and G12-scFv. This result was consistent with their serum concentration, terminal elimination half-life (*t*_1/2_), and EC_50_ against HBsAg. The efficacy of mRNA (G12)-LNPs on HBsAg serum clearance was highly correlated with serum concentration level of synthesized G12 antibodies on the first 2 days, with efficacy ranking as mL (G12-scFv-Fc), mL (G12-IgG), and mL (G12-scFv). In addition, mL (G12-IgG) and mL (G12-scFv-Fc) provided comparable efficacy in HBsAg reduction from day 9 to day 30, although the serum concentration level of synthesized G12-scFv-Fc was higher in the mL (G12-scFv-Fc) group than that in the mL (G12-IgG) group, consistent with the highest affinity of IgG antibodies to HBsAg.

In serum, the antibody concentration provided by mRNAs was much lower than that of exogenous antibodies. The mRNA must be translated by the ribosome for antibody production, so there was a delay before mRNA became effective after administration. Although they arose later than exogenous antibodies, mRNA (G12)-LNPs exerted more durable HBsAg and HBV DNA suppression *in vivo*. Consequently, mRNA drugs were more effective against HBV than G12 antibodies in a mouse model. The result indicated that mRNA-LNP provided a potent adjuvant activity for passive immunization, which may benefit HBsAg clearance. mRNA molecules could be detected by Toll-like receptor 7 (TLR7) and TLR8 (34). Also, the lipid component of the mRNA-LNP platform used in preclinical vaccine studies is highly inflammatory (35). Many studies focus on decreasing mRNA’s Toll recognition by nucleoside modification to balance protein expression and immunostimulatory properties (8). With the help of nucleoside modification, G12 antibody-encoding mRNA-LNPs still had a strong immunostimulatory function, which may further strengthen the passive immunization induced by G12 antibodies. Correspondingly, mRNA-LNPs may alleviate immune tolerance of HBV infection by TLR7/8 recognition. In this case, the efficacy of G12 antibody-encoding mRNA-LNPs was better than that of the antibody. Still, more investigations need to be conducted for the underlying mechanism.

To achieve a functional cure of CHB, G12 antibody-encoding mRNA-LNPs were insufficient. The “sandwich” strategy combines an anti-HBsAg neutralizing antibody with an antiviral drug followed by vaccines for specific active immunization (36). Shi et al. have proven that this strategy has the potential to achieve a functional cure for HBV infection (26). The potential application of mL (G12-IgG) in the combination therapy for HBV infection was underlined by its sustained and progressive effect on reducing HBsAg levels. Thus, anti-HBsAg antibody-encoding mRNA-LNP could be a more potent alternative for anti-HBsAg antibody in the sandwich strategy. Moreover, LNP technology tends to deliver RNA to hepatocytes, which makes it an ideal platform for treating hepatic diseases (28). In addition, LNP application avoids the complex processes and huge cost of antibody production (37, 38). Overall, mRNA therapeutics are promising for treating infectious diseases because of their rapid development, economic value, and simplicity.

## MATERIALS AND METHODS

### Plasmid construction and protein expression.

The human IgG1 monoclonal antibody G12 against HBsAg was produced as reported previously (25).

The G12-scFv gene was constructed with the G12-IgG VL domain, a (G_4_S)_3_ linker, and the G12-IgG VH domain. Then, the gene was cloned into the pComb3x vector with the N-terminal signal peptide (MKKTAIAIAVALAGFATVAQA) and two C-terminal tags (His6 and FLAG). After the G12-scFv gene sequence was validated, a single fresh colony transformed by HB2151 cells was added to 2 mL of 2× yeast extract tryptone [2YT]) medium (100 μg/mL ampicillin and 2% [wt/vol] glucose), shaken at 250 rpm at 37°C for 4 h, and then transformed with 500 mL of superbroth (SB) medium (100 μg/mL ampicillin). When the optical density at 600 nm (OD_600_) of culture media reached 0.6 to 0.8, the culture media were incubated overnight at 30°C at 250 rpm with 1 mM isopropyl-1-thio-β-d-galactopyranoside (IPTG). Bacteria were collected at 8,000 rpm for 10 min and lysed by polymyxin B (Sigma-Aldrich) at 30°C for 0.5 h. The supernatant was generated by centrifuging at 8,000 rpm for 10 min and then loaded over nickel-nitrilotriacetic acid resin according to the manufacturer's instructions (GE Healthcare).

The G12-scFv-Fc gene (synthesized by Azenta Life Sciences), composed of G12-scFv and human IgG1 Fc, was subcloned into the pSecTag 2B vector. Once the sequence was confirmed, the G12-scFv-Fc gene was transfected to Expi293 by polyethyleneimine (PEI; Thermo Fisher). After 6 days, supernatants were harvested at 2,500 rpm for 15 min and filtered with a 0.22-μm vacuum filter. The recombinant protein was purified using protein G resin (GE Healthcare). Protein purity was estimated by SDS-PAGE, and protein concentration was measured by the NanoDrop 2000 spectrophotometer (Thermo Fisher).

### Preparation of mRNAs.

The mRNAs were synthesized using *in vitro* transcription (IVT) by T7 RNA polymerase. In brief, the coding sequence of the G12 antibody and the flanking 5′ and 3′ UTRs were subcloned into the pUC57 vector. The DNA templates for transcription were prepared by PCR. The poly(A) coding sequence was introduced during PCR. The PCR product was used for the subsequent *in vitro* transcription. The cap1 structure was introduced to the 5′ ends of mRNA during transcription using the Cap1 analog (ApexBio; catalog no. B8176). Pseudouridine was used to substitute uridine. Reaction mixtures were incubated at 37°C for 2.5 h, followed by DNase I (NEB; catalog no. M0303) treatment for 30 min. Finally, mRNAs were purified by oligo(dT) affinity chromatography.

### Preparation and characteristics of mRNA LNPs.

LNP formulation was prepared as previously described (10). The lipids were dissolved in ethanol at molar ratios of 50:10:38.5:1.5 (SM-102/1,2-distearoyl-*sn*-glycero-3-phosphocholine [DSPC]/cholesterol/DMG-PEG2000. mRNA was diluted in 50 mM citrate buffer (pH 4.0). For G12-IgG production, LC mRNA and HC mRNA were mixed in the same mRNA solution at the mass ratio of 1:2 (LC mRNA to HC mRNA). The lipid mixture was mixed with mRNA solution at the volume ratio of 1:3 using a microfluidic mixer (Precision NanoSystems). The N/P ratio was about 6:1. LNPs were immediately diluted with an equal volume of 50 mM citrate buffer (pH 4.0) and then dialyzed for about 16 h. Using Amicon ultracentrifugal filters (Millipore), formulations were concentrated and passed through a 0.22-μm filter. All LNP formulations were characterized for particle size, RNA encapsulation, and endotoxin. The size of the particle was characterized using Zetasizer Nano ZS ZEN3600 (Malvern Instruments Limited). mRNA encapsulation efficiency was determined by the Quant-it RiboGreen RNA assay (Thermo Scientific).

### mRNAs expression in cell lines.

293T cells were cultured in high-glucose Dulbecco’s modified Eagle medium (DMEM; Gibco) supplemented with 10% fetal bovine serum (FBS) and 1% penicillin-streptomycin (P/S) (Gibco) in 5% CO_2_ at 37°C. Cells were seeded at a density of 2 × 10^5^ cells per well on a 12-well plate. mRNAs were transfected by Lipofectamine 2000 (Thermo) at a ratio of 1:1.5 (mRNA/Lipofectamine 2000) for 6 h and kept incubated for another 18 h until detection.

### Pharmacokinetic profile.

C57 mice were divided into groups (5 mice per group). Mice were injected i.v. with mRNA-LNPs at a dose of 2.5 mg/kg or antibodies at 6.7 mg/kg. Blood samples were then taken from the periorbital venous sinus at indicated time points (0.25, 0.5, 1, 3, 6, 12, 24, 48, 72, 96, and 168 h) and centrifuged at 4,000 rpm for 10 min for the plasma collection.

### AAV/HBV mouse models and anti-HBV therapies.

C57 mice were injected i.v. with 2 × 10^10^ AAV/HBV1.3 (Five-plus Molecular Medicine Institute, China). After 2 weeks, an AAV/HBV mouse model (39) was established. C57 mice were divided into groups (4 mice per group). Mice were injected i.v. with mRNA-LNPs at a dose of 2.5 mg/kg or antibodies at 6.7 mg/kg. Blood samples were taken from the periorbital venous sinus after treatment at indicated time points (1, 2, 5, 9, 12, 15, 20, 26, and 30 days). Blood was collected and centrifuged at 4,000 rpm at room temperature for 10 min. Plasma was collected and stored at −80°C until analysis.

### Enzyme-linked immunosorbent assay (ELISA).

Briefly, plates (Corning; catalog no. 3690) were coated with 100 ng/well HBsAg (North China Pharmaceuticals Co., Ltd.) in phosphate-buffered saline (PBS) at 4°C overnight and blocked with 3% milk (wt/vol) diluted in PBS at 37°C for 1 h. Cell supernatant or plasma was serially diluted in 1% bovine serum albumin (BSA) buffer. Plates were washed 3 times in PBS-T (0.05% Tween 20 in PBS) and then incubated with diluted samples at 37°C for 1.5 h. Then, plates were incubated with secondary antibody, horseradish peroxidase (HRP)-conjugated anti-human IgG Fc antibody (Thermo Fisher) for IgG antibody and Fc recombinant protein, and mouse anti-His antibody (GenScript) at 37°C for scFv antibody at 37°C for 45 min after washing for 5 times in PBS-T. Plates were washed 5 times and then incubated with ABTS [2,2′-azinobis(3-ethylbenzthiazolinesulfonic acid)] at 37°C for 15 min. Analysis was quickly conducted at wavelength of 405 nm by a microplate reader (BioTek).

### Detection of HBV markers.

HBsAg levels were rationed by the Roche cobas 6000 (Shanghai Labway Clinical Laboratory Co., Ltd.). The HBV DNA levels were quantified by the HBV DNA quantitative fluorescent diagnostic kit (Shengxiang Co., Ltd., China).

### Statistical methods.

All data were analyzed using GraphPad Prism 8 (CA, USA). Statistical analysis was performed by unpaired *t* test and one-way analysis of variance (ANOVA). The results were shown as mean ± standard deviation (SD) (*n *≥ 3). Statistically, significant differences were defined as *, *P < *0.05; **, *P < *0.01; ***, *P < *0.001; and ****, *P < *0.0001.

### Ethics statement.

All the procedures related to animal handling, care, and treatment were performed and approved by the Ethics Committee of the School of Basic Medical Sciences at Fudan University according to the recommendations in the Guide for the Care and Use of Laboratory Animals, Fudan University.
