# Supplementary figures and images for: A Single Dose of Anti-HBsAg Antibody-Encoding mRNA-LNPs Suppressed HBsAg Expression: a Potential Cure of Chronic Hepatitis B Virus Infection
Source: mBio. 2022 Jul 7;13(4):e01612-22. doi: 10.1128/mbio.01612-22 (PMC9426588; doi:10.1128/mbio.01612-22)

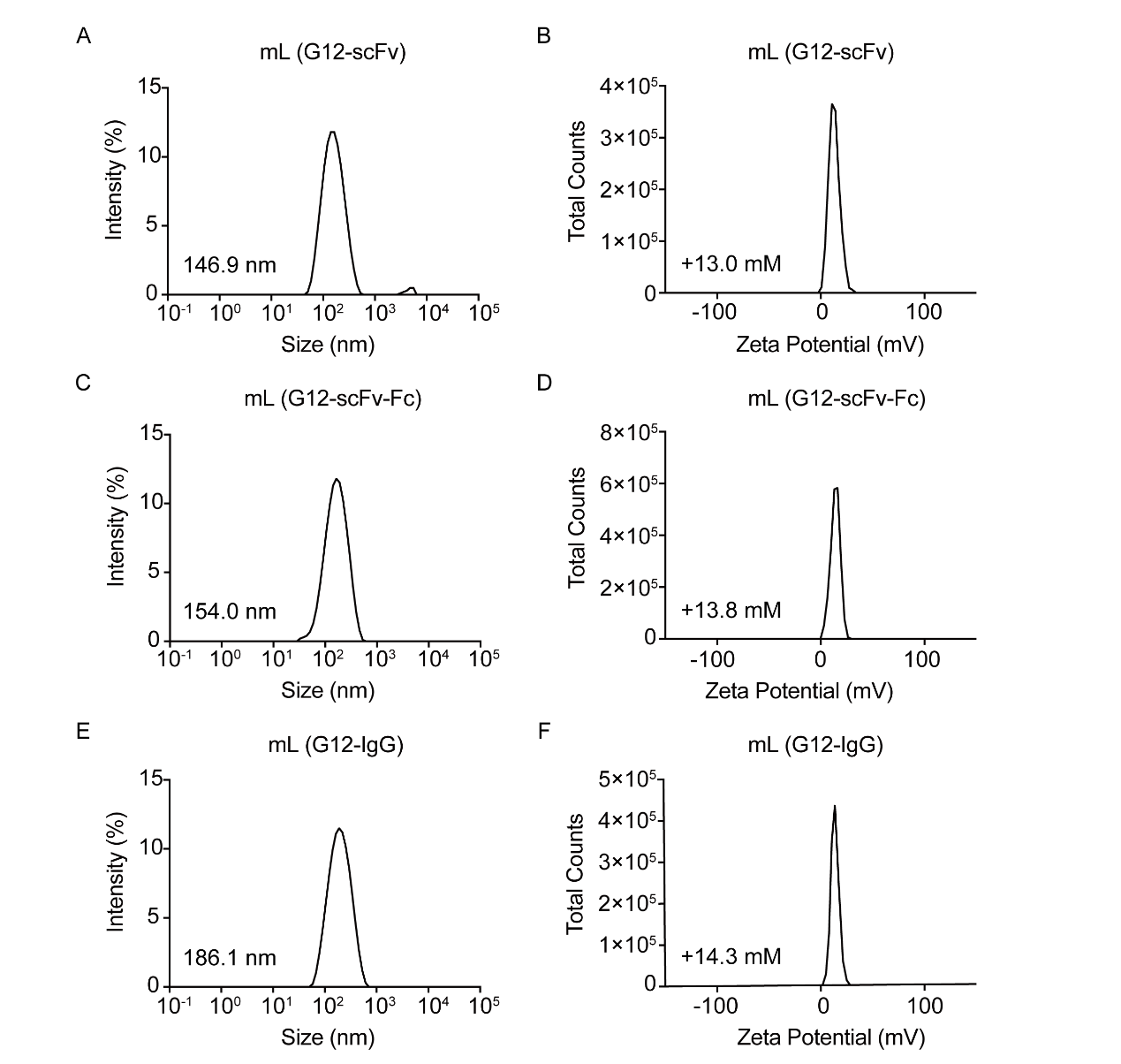

Supplement: FIG S1 [file mbio.01612-22-s0001.tif]

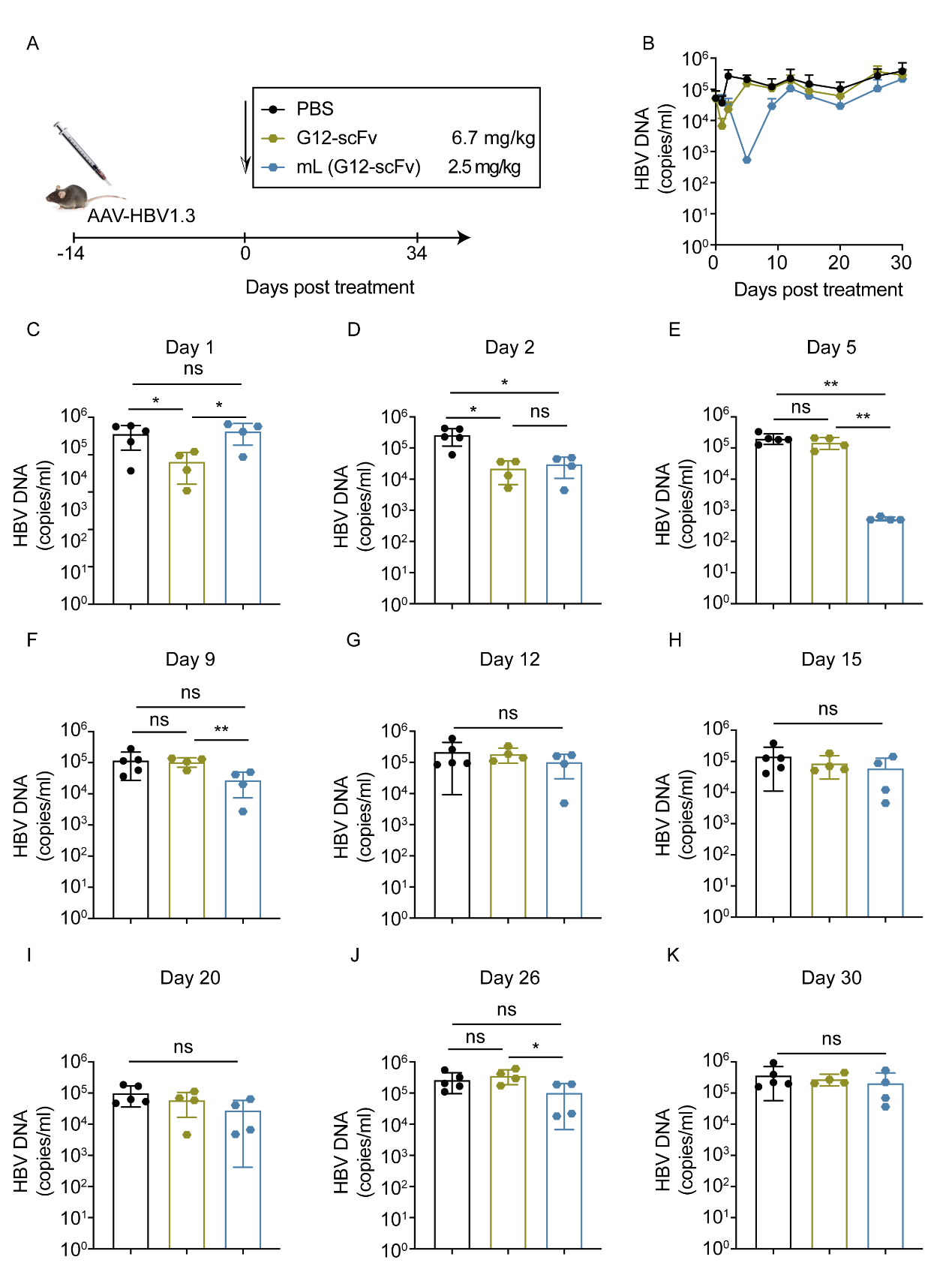

Supplement: FIG S2 [file mbio.01612-22-s0002.tif]

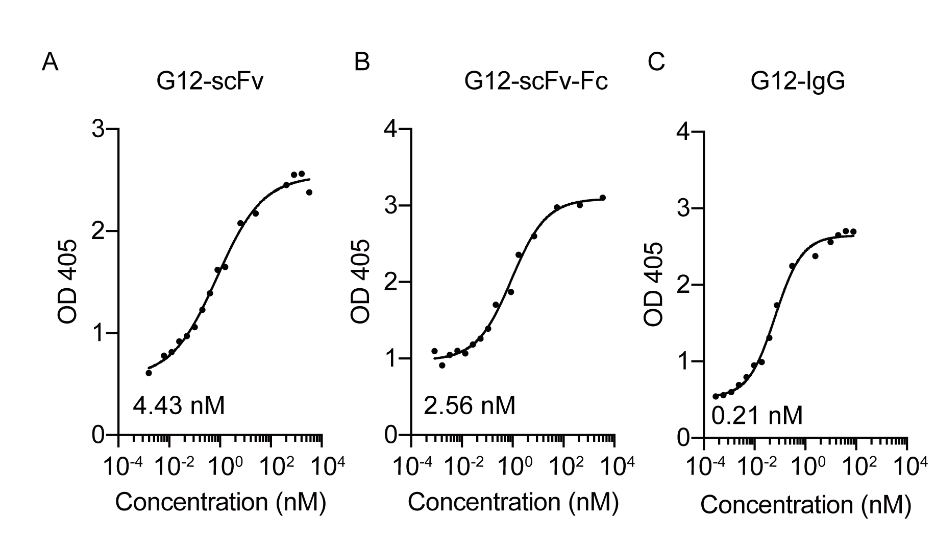

Supplement: FIG S5 [file mbio.01612-22-s0005.tif]

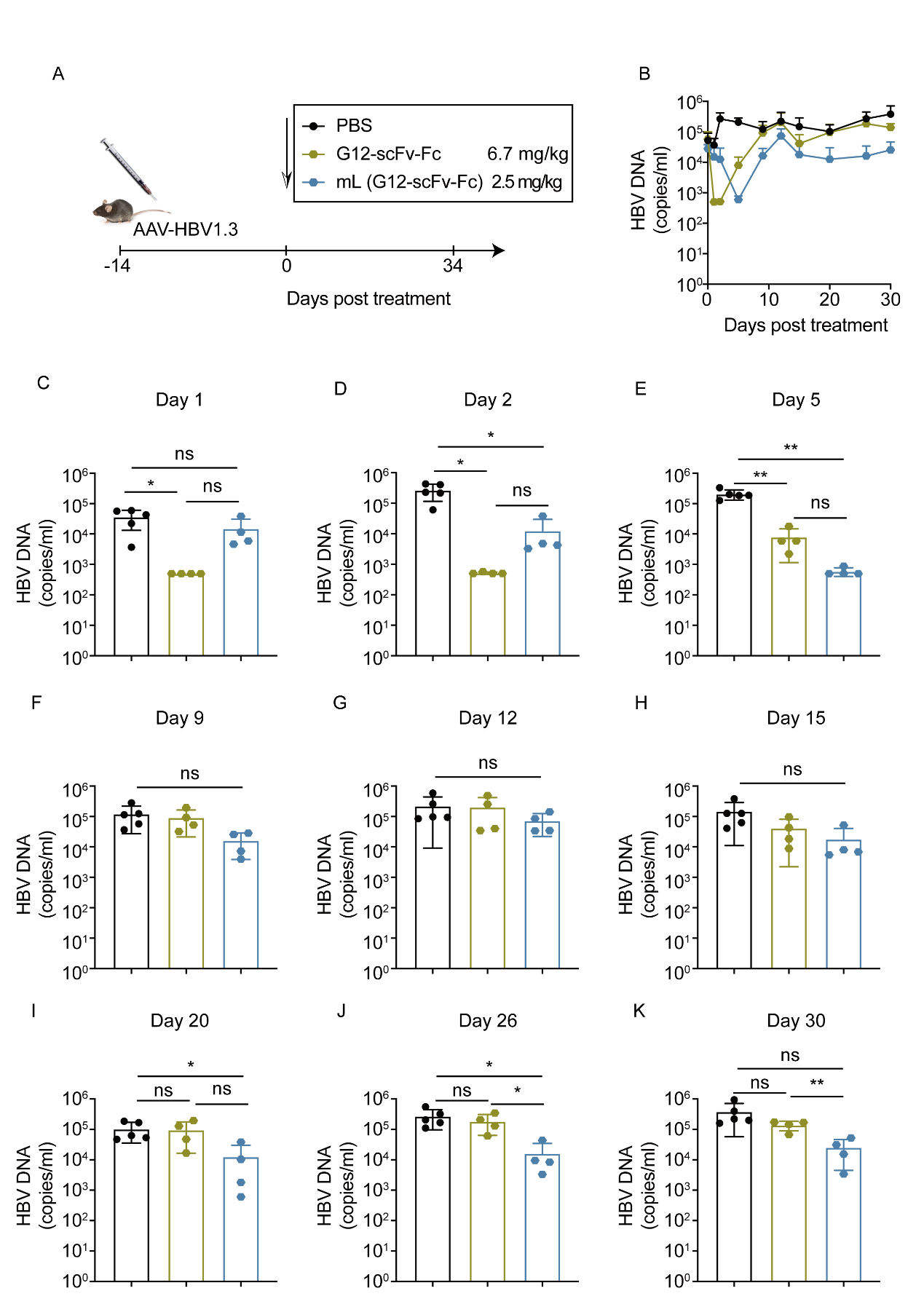

Supplement: FIG S3 [file mbio.01612-22-s0003.tif]

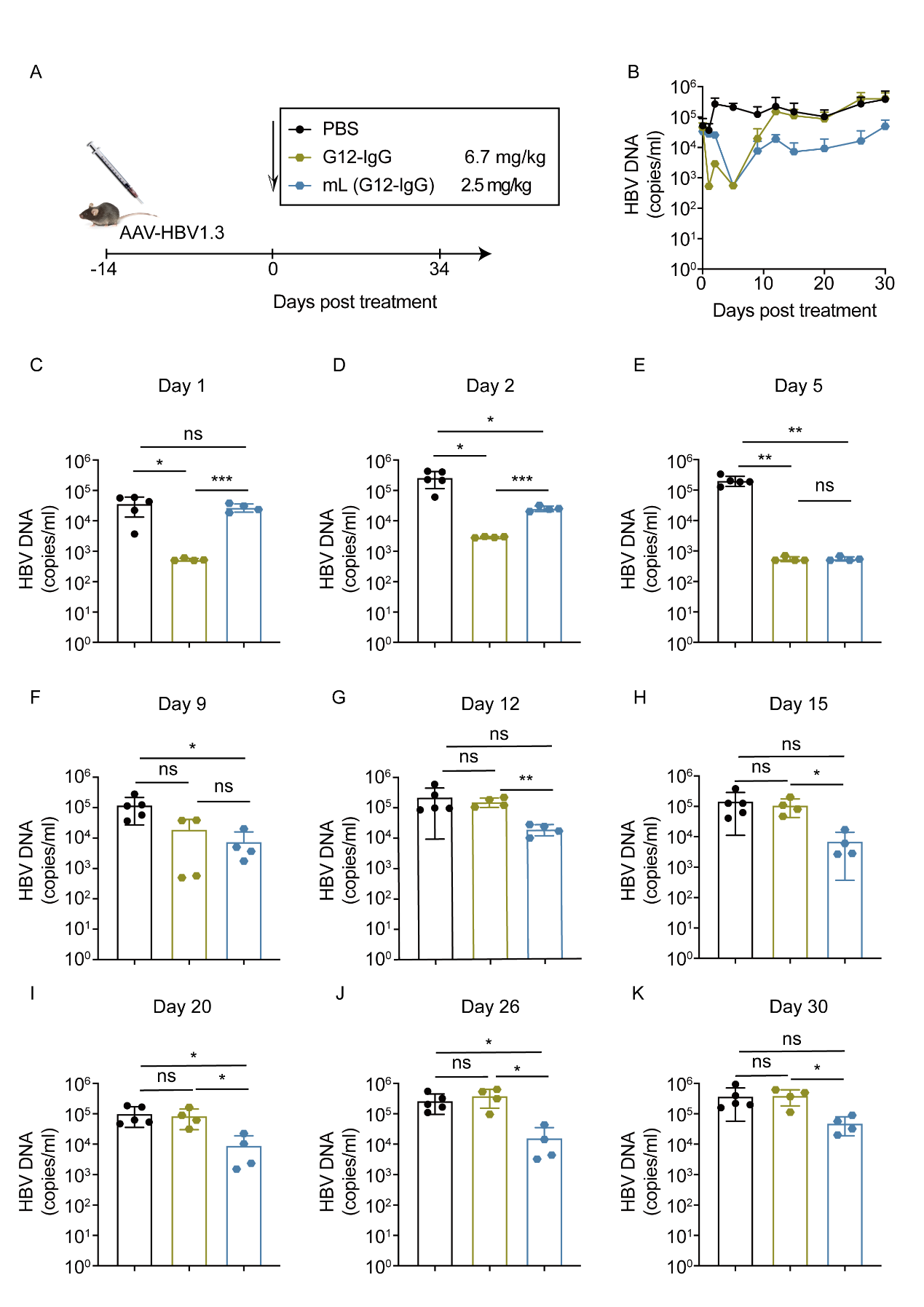

Supplement: FIG S4 [file mbio.01612-22-s0004.tif]
